# Mucormycosis in South America: A review of 143 reported cases

**DOI:** 10.1111/myc.12958

**Published:** 2019-07-11

**Authors:** Marcio Nucci, Marc Engelhardt, Kamal Hamed

**Affiliations:** ^1^ Department of Internal Medicine, University Hospital Universidade Federal do Rio de Janeiro Rio de Janeiro Brazil; ^2^ Basilea Pharmaceutica International Ltd. Basel Switzerland

**Keywords:** antifungal agents, Brazil, invasive fungal disease, mucormycosis, South America, systemic infection

## Abstract

Mucormycosis is a rare but important invasive fungal disease that most often affects immunocompromised hosts. The incidence of mucormycosis appears to be increasing worldwide, as risk factors such as the use of immunosuppressive therapies become more common. We report the results of a literature review of 143 mucormycosis cases reported in South America between 1960 and 2018. The number of reported cases has increased by decade, from 6 in the 1960s to 51 in the 2010s. The most common underlying conditions associated with mucormycosis in South America were diabetes mellitus (42.0%) and penetrating trauma/burns (20.0%). Underlying conditions involving immunosuppression, including treatment of haematologic malignancy, solid organ transplant, and corticosteroid use, also accounted for a large proportion of cases (45.5%). Between 1960 and 2018, cases of mucormycosis associated with conditions involving immunosuppression accounted for the highest mortality rate (58.5%), followed by diabetes mellitus (45.0%), and penetrating trauma/burns (37.9%). Overall mortality decreased from 100% to 39.4% during this period, mainly driven by the increasing availability and use of antifungal therapies and surgical intervention. However, these treatments are not yet universally utilised across the region in the treatment of mucormycosis; efforts to improve availability of effective treatments would be likely to improve outcomes.

## 1 INTRODUCTION

Invasive fungal disease (IFD) is an important complication in immunocompromised patients.^1^, ^2^ The frequency of IFD has increased in correlation with growth in populations of immunocompromised hosts, including patients with HIV infection, cancer, organ transplantation and autoimmune diseases.^1^, ^2^

Mucormycosis has emerged as a rare but important IFD, affecting patients worldwide. The disease is associated with high morbidity and mortality.^3^, ^4^, ^5^, ^6^, ^7^ An increase in the incidence of mucormycosis has been seen in recent decades,^8^, ^9^, ^10^, ^11^ as exemplified by the 7.3% yearly increase observed in France between 2001 and 2010.^9^

Mucormycosis is caused mainly by fungi of the Mucorales order, with >25 species having been reported to infect humans.^12^ Historically, the terms mucormycosis and zygomycosis (the term for diseases caused by fungi of the Zygomycota phylum, which included Mucorales and Entomophthorales) were used interchangeably to refer to these diseases. Infection due to Entomophthorales is typically localised.^3^, ^5^ This review focuses only on mucormycosis.

Mucormycosis may be acquired through different routes, including the respiratory tract, injured skin, contaminated needles or catheters, or ingestion of contaminated food.^7^ The most common sites of infection are the rhino‐orbital‐cerebral, pulmonary, gastrointestinal and cutaneous areas.^4^, ^11^, ^13^ Following the initial infection, the disease typically progresses quickly, with rapid invasion of blood vessels leading to thrombosis and tissue necrosis.^3^, ^7^

A number of different factors are associated with an increased risk of mucormycosis.^5^, ^10^ Globally, mucormycosis is particularly common in patients with diabetes mellitus, and the risk is much higher in patients with uncontrolled diabetes, in whom the resultant ketoacidosis interferes with the normal activity of lymphocytes, increasing risk of infection.^3^, ^4^, ^6^, ^7^, ^14^, ^15^, ^16^ In addition, as with other IFD, patients with an impaired immune system following the use of immunosuppressive treatments are also vulnerable. This group includes patients receiving chemotherapy as treatment for cancer, especially haematological malignancies, and also hematopoietic stem cell transplantation (HSCT) and solid organ transplant (SOT) recipients.^3^, ^4^, ^6^, ^7^, ^16^ Mucormycosis also affects immunocompetent individuals after penetrating trauma or burns, which exposes tissues to environmental sources of fungi, and patients with iron overload under treatment with deferoxamine.^3^, ^4^, ^6^, ^7^, ^16^

Characteristically, fungi belonging to the order Mucorales exhibit high minimum inhibitory concentrations (MICs) to many antifungal agents currently available.^12^ The antifungal agents with the lowest MICs for Mucorales are amphotericin B, isavuconazole and posaconazole, but the MICs vary widely depending on the genus and species. In the clinical setting, MICs of each antifungal agent for each genus and species need to be interpreted in the context of the antifungal exposures that are achievable.^17^ Prompt diagnosis and rapid initiation of antifungal therapy combined with surgical removal of infected tissue are required for optimal outcomes. In addition, control of the underlying condition and/or reduction of immunosuppression are important components of treatment.^3^, ^12^, ^13^, ^14^

As acknowledged by the European Confederation of Medical Mycology (ECMM), there are currently regional differences in the diagnosis and treatment of mucormycosis. As such, the ECMM has recently begun a “neglected orphan disease guidance initiative” focusing on this disease both within and beyond the European region.^18^ Several previous studies have gathered data on mucormycosis in patients in Europe, Asia or other areas,^3^, ^4^, ^5^, ^8^, ^9^, ^10^, ^11^, ^19^, ^20^ but to date, there has been no comprehensive review of the literature on mucormycosis in South America. Here, we collate and review mucormycosis case studies reported in South American countries, exploring patient characteristics, course of infection, treatment regimens and clinical outcomes.

## 2 METHODS

We performed a PubMed and Embase search of the medical literature through October 2018 using the following keywords: mucormycosis, mucor*, zygomycos*, zygomycet*, zigomicos*, zigomicet*, cigomicos*, cigomicet*, phycomycos*, ficomicos*, phicomicet*, ficomicet*, Mucorales, *Rhizopus*, *Mucor*, *Rhizomucor*, *Saksenaea*, *Apophysomyces*, *Cunninghamella*, *Lichtheimia*, *Absidia*, *Syncephalastrum*, plus the names of the individual South American countries. There were no language restrictions. Infections documented to be caused by fungi of the order Entomophthorales were excluded.

The publications identified in the search were screened manually to identify publications that included relevant information based on their abstracts and/or full text. We only included case reports for which all of the following information was described for each individual patient: underlying condition(s), site of infection, documentation of infection (histological or by culture), therapeutic intervention (presence or absence of antifungal therapy and surgery), type of antifungal therapy (if any) and outcome. Mortality was included regardless of cause.

Data were entered in Microsoft^®^ Excel^®^ 2010 and analysed using descriptive statistics only. Duplicates were identified within Excel and removed manually. Redundant cases appearing in more than one journal article were eliminated. Case series that summarised data across patients instead of reporting these data separately for each individual patient were excluded. Three such publications, all from Chile, were identified.^21^, ^22^, ^23^

The authors confirm that the ethical policies of the journal, as noted on the journal's author guidelines page, have been adhered to. No ethical approval was required as this is a review article with no original research data.

## 3 RESULTS

Literature searches identified cases of mucormycosis in 59 patients from Brazil and 84 from the wider South America region (Argentina, 36; Chile, 14; Colombia, 22; Venezuela, 7; Peru, 3; and Ecuador and French Guiana, 1 case each), with a total of 143 cases. Full reference lists are provided in the Supporting information. As around 41% of cases were from a single country, Brazil, these cases are presented separately to avoid skewing the results for the whole region in favour of a single country.

**Figure 1:**
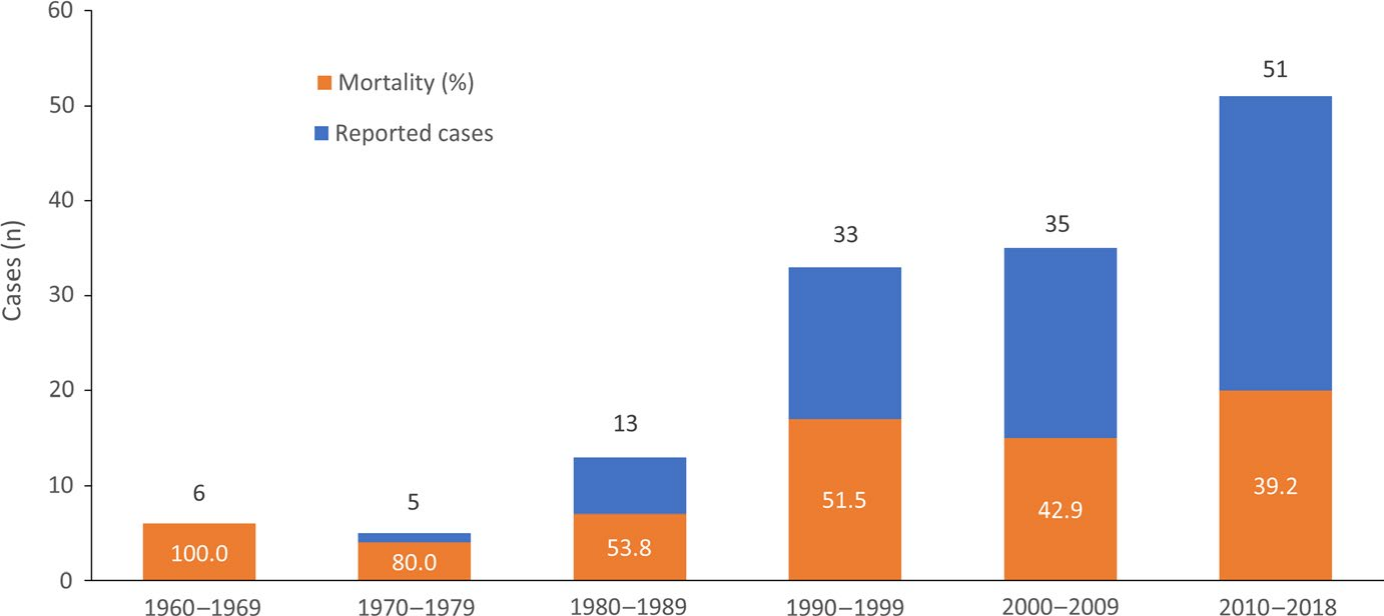
Number of published cases of invasive mucormycosis reported in South America by decade since 1960, and corresponding mortality

The earliest case was reported in April 1965,^24^ and the latest case was reported in July 2018.^25^ Only five cases were reported in the literature in the period between 1970 and 1979, but the number of published cases has increased with each subsequent decade, with 51 already published within the current decade. The number of deaths related to mucormycosis has also increased in parallel, but the overall mortality rate across all cases appears to have decreased (Figure 1).

The median age of the patients was 40.0 years (43.0 in Brazil, 37.5 in the other countries); the age distribution of patients was as follows: <1‐17 years, 25 patients; 18‐44 years, 55 patients; 45‐64 years, 52 patients; 65‐84 years, 10 patients; ≥85 years, one patient. The majority of patients (61.5%) were male (62.7% in Brazil, 60.7% in the other countries). Demographics and details of patients’ underlying conditions are provided in Table 1.

**Table 1:** Demographic and clinical characteristics and associated mortality among identified cases of mucormycosis in South America

	Brazil		Other South American countries		Total	
	Characteristic (N = 59)	Mortality n (%)^a^	Characteristic (N = 84)	Mortality n (%)^a^	Characteristic (N = 143)	Mortality n (%)^a^
Age, y						
Mean	39.6	NA	39.1	NA	39.3	NA
Median (range)	43.0 (<1‐78)	NA	37.5 (<1‐86)	NA	40.0 (<1‐86)	NA
Sex, n (%)						
Male	37 (62.7)	21 (56.8)	51 (60.7)	23 (45.1)	88 (61.5)	44 (50.0)
Female	22 (37.3)	10 (45.5)	31 (36.9)	14 (45.2)	53 (37.1)	24 (45.3)
Unknown	0 (0)	NA	2 (2.4)	1 (50.0)	2 (1.4)	1 (50.0)
Underlying condition, n (%)^b^						
Diabetes mellitus	25 (42.4)	12 (48.0)	35 (41.7)	15 (42.9)	60 (42.0)	27 (45.0)
Penetrating trauma/burns	2 (3.4)	1 (50.0)	27 (32.1)	10 (37.0)	29 (20.3)	11 (37.9)
Immunosuppression	39 (66.1)	25 (64.1)	26 (31.0)	13 (50.0)	65 (45.5)	38 (58.5)
Malignancy	10 (16.9)	6 (60.0)	9 (10.7)	5 (55.6)	19 (13.3)	11 (57.9)
Haematologic	7 (11.9)	3 (42.9)	9 (10.7)	5 (55.6)	16 (11.2)	8 (50.0)
Allogeneic HSCT	3 (5.1)	3 (100.0)	0 (0)	NA	3 (2.1)	3 (100.0)
Solid organ transplant	13 (22.0)	9 (69.2)	5 (6.0)	2 (40.0)	18 (12.6)	11 (61.1)
Use of corticosteroids	6 (10.2)	4 (66.7)	3 (3.6)	1 (33.3)	9 (6.3)	5 (55.6)
Chronic pulmonary disease	1 (1.7)	1 (100.0)	0 (0)	NA	1 (0.7)	1 (100.0)
Chronic renal failure	1 (1.7)	1 (100.0)	0 (0)	NA	1 (0.7)	1 (100.0)
Other	8 (13.6)	5 (62.5)	4 (4.8)	4 (100.0)	12^c^ (8.4)	9 (75.0)
None (at time of initial infection)	6 (10.2)	1 (16.7)	5 (6.0)	2 (40.0)	11 (7.7)	3 (27.3)

Abbreviations: AIDS, acquired immune deficiency syndrome; HIV, human immunodeficiency virus; HSCT, hematopoietic stem cell transplant; NA, not applicable.

a Percentages are numbers of patients with the characteristic who died/total numbers with the characteristic.

b Patients may have more than one underlying condition.

c Includes malnutrition (n = 4), HIV infection/AIDS (3), bone marrow aplasia (1), liver cirrhosis (1), ankylosing spondylitis (1), thalidomide therapy for leprosy (1) and prematurity (1).

The most prevalent underlying condition in Brazil and in the other countries was diabetes mellitus (42.4% and 41.7%, respectively). Of note, the proportion of cases caused by diabetes remained stable by decade (1960‐69, 50.0%; 1970‐79, 37.5%; 1980‐89, 53.8%; 1990‐99, 27.3%; 2000‐09, 48.6%; 2010‐18, 41.2%).

After diabetes mellitus, the next most common conditions were SOT (22.0%) and malignancy (16.9%) in Brazil, and penetrating trauma/burns (32.1%) and malignancy (10.7%) in the other countries. Of note, the incidence of penetrating trauma/burns as an underlying cause was much lower in Brazil than in the other countries (3.4% vs 32.1%). Thirteen of the 29 cases of penetrating trauma were from Colombia, and eight of these cases were skin and soft tissue infection after a volcanic eruption.^26^ The overall mortality rate was 48.3%, 52.5% in Brazil and 45.2% in the other countries. The mortality rate in cases secondary to penetrating trauma/burns was lower than in cases occurring in immunocompromised patients (20.3% and 45.5%, respectively). Of three patients who received an allogeneic HSCT in Brazil, all three died (Table 1).

In Brazil, the most frequent sites of infection (≥10% of patients) were rhino‐sino‐orbito‐cerebral in 22 patients (37.3%) and pulmonary in 14 patients (23.7%). In the other countries, the most frequent sites of infection were rhino‐sino‐orbito‐cerebral in 42 patients (50.0%) and skin and soft tissues in 28 patients (33.3%). Mortality rates throughout the region were >30% for all infection sites except liver/kidneys and were particularly high (>60%) in disseminated, gastrointestinal/peritoneum and pulmonary infections (Table 2).

**Table 2:** Sites of infection and associated mortality among identified cases of mucormycosis in South America

Site of infection	Brazil		Other South American countries		Total	
	Incidence (N = 59) n (%)	Mortality n (%)^a^	Incidence (N = 84) n (%)	Mortality n (%)^a^	Incidence (N = 143) n (%)	Mortality n (%)^a^
Rhino‐sino‐orbito‐cerebral	22 (37.3)	10 (45.5)	42 (50.0)	20 (47.6)	64 (44.8)	30 (46.9)
Pulmonary	14 (23.7)	10 (71.4)	3 (3.6)	1 (33.3)	17 (11.9)	11 (64.7)
Skin and soft tissues	6 (10.2)	0 (0)	28 (33.3)	11 (39.3)	34^b^ (23.8)	11 (32.4)
Gastrointestinal or peritoneum	4 (6.8)	3 (75.0)	1 (1.2)	1 (100.0)	5 (3.5)	4 (80.0)
Liver or kidneys	3 (5.1)	0 (0)	0 (0)	NA	3 (2.1)	0 (0)
Other	3 (5.1)	2 (66.7)	5 (6.0)	1 (20.0)	8^c^ (5.6)	3 (37.5)
Disseminated infection^d^	7 (11.9)	6 (85.7)	5 (6.0)	4 (80.0)	12 (8.4)	10 (83.3)

Abbreviation: NA, not applicable.

a Percentages are numbers of patients with the site of infection who died/total numbers with the site of infection.

b Includes lower extremity (n = 10), trunk (10), upper extremity (5), head/face (5), lower extremity and trunk (2) and unknown (2).

c Includes bone (4), central nervous system (2), endophthalmitis after cataract surgery (1) and oral cavity (1).

d More than one non‐contiguous site.

**Figure 2:**
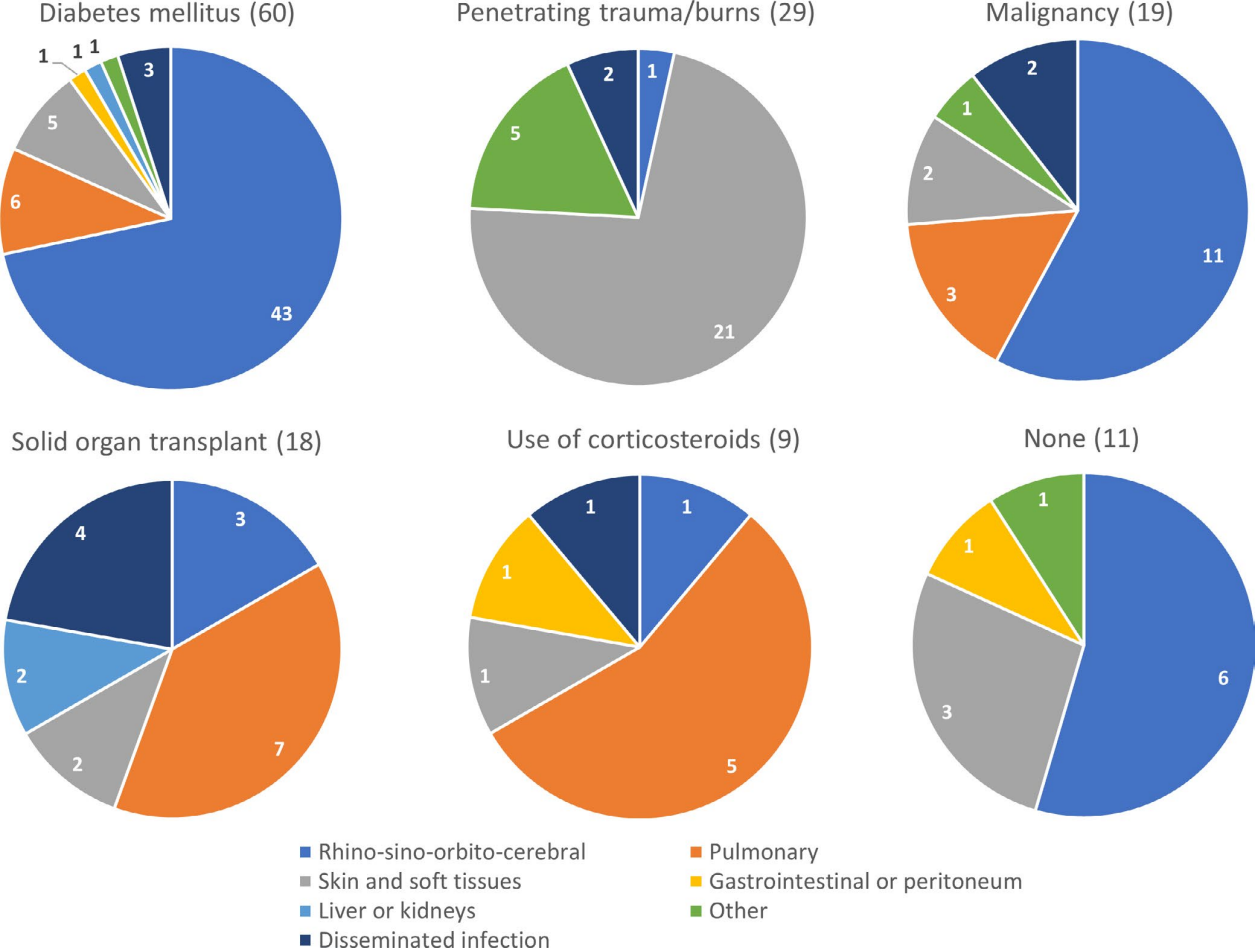
Number of reported mucormycosis cases by site, separated according to underlying disease

In South America overall, diabetes mellitus was mostly associated with rhino‐sino‐orbito‐cerebral infection (43/60 cases; 71.7%), as was malignancy (11/19 cases; 57.9%). Episodes of penetrating trauma or burns were mostly associated with skin and soft tissue infection (21/29 cases), and corticosteroid use was mostly associated with pulmonary infections (5/9 cases; Figure 2).

No pathogenic species was identified in 35 cases in Brazil (59.3%) and 37 cases in other countries (44.0%). In cases with identification of genus (24 in Brazil; 47 in the other countries), *Rhizopus* (11 cases in Brazil; 27 cases outside Brazil), followed by *Mucor* (7 cases in Brazil; 10 cases outside Brazil), were the most frequent. Details of the species identified are given in Table 3.

**Table 3:** Mycological findings among identified cases of mucormycosis in South America

Fungal organism	Brazil		Other South American countries		Total	
	Incidence (N = 59) n (%)	Mortality n (%)^a^	Incidence (N = 84) n (%)	Mortality n (%)^a^	Incidence (N = 143) n (%)	Mortality n (%)^a^
*Rhizopus* species	11 (18.6)	9 (81.8)	27 (32.1)	9 (33.3)	38 (26.6)	18 (47.4)
*R arrhizus*	8 (13.6)	7 (87.5)	12 (14.3)	6 (50.0)	20 (14.0)	13 (65.0)
*R microsporus*	2 (3.4)	2 (100.0)	4 (4.8)	0 (0)	6 (4.2)	2 (33.3)
Not speciated	1 (1.7)	0 (0)	11 (13.1)	3 (27.3)	12 (8.4)	3 (25.0)
*Mucor* species	7 (11.9)	2 (28.6)	10 (11.9)	3 (30.0)	17 (11.9)	5 (29.4)
*M hiemalis*	2 (3.4)	0 (0)	0 (0)	NA	2 (1.4)	0 (0)
*M indicus*	0 (0)	NA	1 (1.2)	0 (0)	1 (0.7)	0 (0)
Not speciated	5 (8.5)	2 (40.0)	9 (10.7)	3 (33.3)	14 (9.8)	5 (35.7)
*Saksenaea* species	0 (0)	NA	5 (6.0)	1 (20.0)	5 (3.5)	1 (20.0)
*S erythrospora*	0 (0)	NA	2 (2.4)	0 (0)	2 (1.4)	0 (0)
*S vasiformis*	0 (0)	NA	3 (3.6)	1 (33.3)	3 (2.1)	1 (33.3)
*Rhizomucor* species	2 (3.5)	2 (100.0)	1 (1.2)	0 (0)	3 (2.1)	2 (66.7)
*R pusillus*	1 (1.7)	1 (100.0)	0 (0)	NA	1 (0.7)	1 (100.0)
Not speciated	1 (1.7)	1 (100.0)	1 (1.2)	0 (0)	2 (1.4)	1 (50.0)
*Apophysomyces* species	0 (0)	NA	3 (3.6)	1 (33.3)	3 (2.1)	1 (33.3)
*A elegans*	0 (0)	NA	2 (2.4)	1 (50.0)	2 (1.4)	1 (50.0)
*A variabilis*	0 (0)	NA	1 (1.2)	0 (0)	1 (0.7)	0 (0)
*Lichtheimia corymbifere*	2 (3.4)	1 (50.0)	0 (0)	NA	2 (1.4)	1 (50.0)
*Actinomucor elegans*	0 (0)	NA	1 (1.2)	0 (0)	1 (0.7)	0 (0)
*Cunninghamela bertholletiae*	1 (1.7)	1 (100.0)	0 (0)	NA	1 (0.7)	1 (100.0)
*Syncephalastrum* species	1 (1.7)	1 (100.0)	0 (0)	NA	1 (0.7)	1 (100.0)
Mucorales (not otherwise specified)	35 (59.3)	15 (42.9)	37 (44.0)	24 (64.9)	72 (50.3)	39 (54.2)

Abbreviation: NA, not applicable.

a Percentages are numbers of patients with the fungal organism who died/total numbers with the fungal organism.

Most patients received treatment with an antifungal agent (n = 49 [83.1%] for Brazil; n = 65 [77.4%] for the other countries; n = 114 [79.7%] overall). Antifungal treatment was combined with surgery in 35.6% (n = 21) of cases in Brazil and in 59.5% (n = 50) of cases in the other countries.

**Figure 3:**
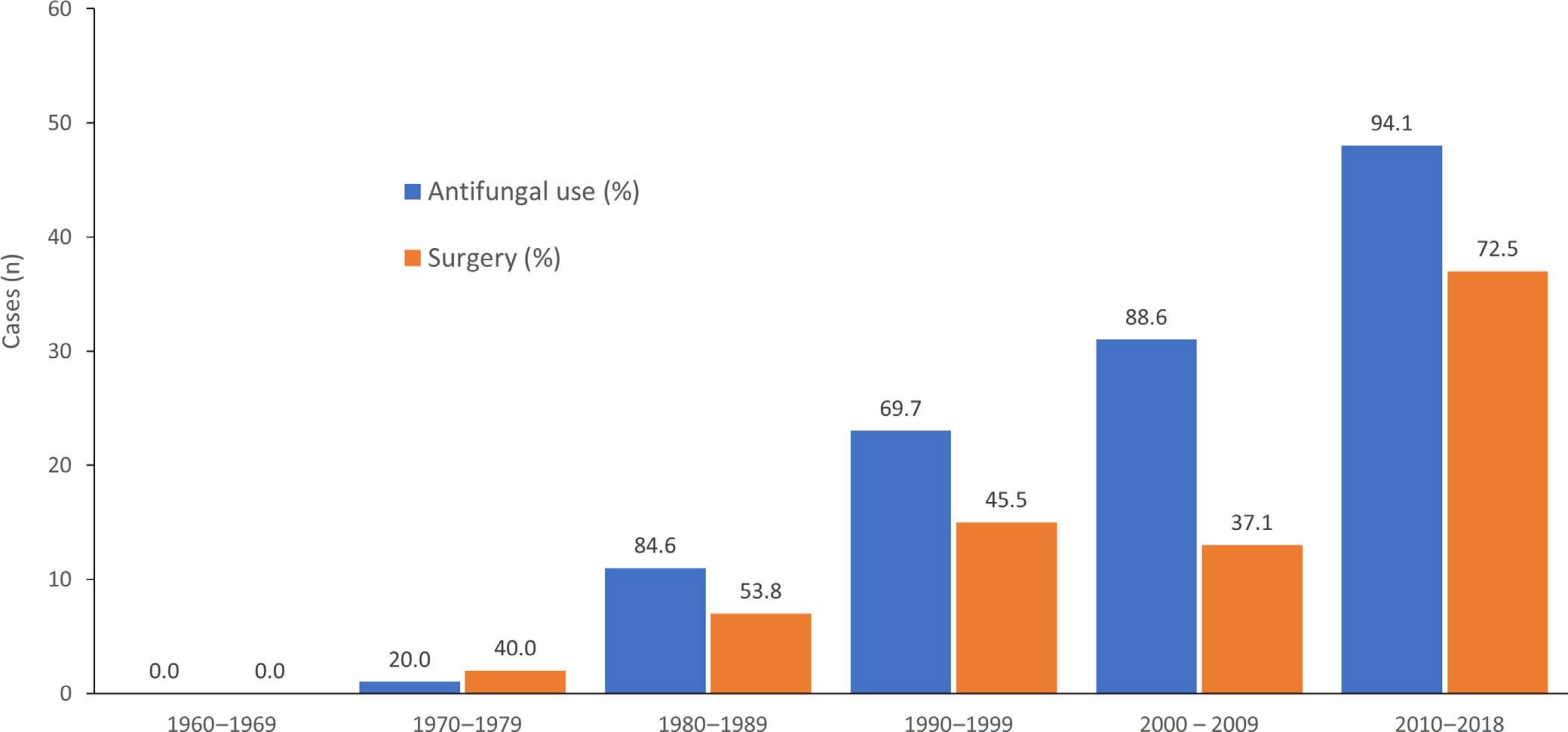
Number and proportion of treatments for published cases of invasive mucormycosis reported in South America by decade since 1960. One patient in 1977 and two patients in 1991 underwent surgery without antifungal treatment

The overall mortality rate with antifungal treatment alone was 58.1%; a lower mortality rate (23.9%) was observed among patients receiving antifungal treatment combined with surgery. As expected, mortality rates were extremely high in patients who did not receive antifungal treatment (10/10 [100%] patients in Brazil; 17/19 [89%] patients in the other countries; Table 4). The two patients who survived without antifungal treatment had developed mucormycosis secondary to penetrating trauma or burns after a volcanic eruption^26^; both patients underwent surgical procedures on their lower extremities. The proportion of patients receiving antifungal treatment combined with surgery among patients with skin and soft tissue infections was higher (64.7%) than among patients with infections at other sites (45.0%). Among patients with non‐skin or soft tissue infection, the lowest rates of surgery were observed in patients with pulmonary infection (1/17 cases; 5.9%) or disseminated infection (2/12 cases; 16.7%) (Table S1). However, a small number of patients with skin and soft tissue infections were reported from Brazil, and so these data are largely from the other countries. Outcomes in patients receiving antifungal treatment combined with surgery were generally similar between patients with skin and soft tissue infections and patients with other sites of infection (approximately 23%‐25% mortality rate) (Table S2). The proportion of patients receiving antifungal treatment increased between 1960 and 2018. A less dramatic increase in the number of surgeries being performed was also observed over time (Figure 3).

**Table 4:** Treatment and outcome among identified cases of mucormycosis in South America

	Brazil		Other South American countries		Total	
	Incidence (N = 59) n (%)	Mortality n (%)^a^	Incidence (N = 84) n (%)	Mortality n (%)^a^	Incidence (N = 143) n (%)	Mortality n (%)^a^
Treatment (overall)						
Antifungal only	28 (47.5)	16 (57.1)	15 (17.9)	9 (60.0)	43 (30.1)	25 (58.1)
Antifungal + surgery	21 (35.6)	5 (23.8)	50 (59.5)	12 (24.0)	71 (49.7)	17 (23.9)
No antifungal treatment	10 (16.9)	10^b^ (100.0)	19 (22.6)	17^c^ (89.5)	29 (20.3)	27 (93.1)
Type of antifungal treatment						
Amphotericin B only	44 (74.6)	18 (40.9)	54 (64.3)	20 (37.0)	98 (68.5)	38 (38.8)
Deoxycholate formulation	39 (66.1)	17 (43.6)	44^d^ (52.4)	19 (43.2)	83 (58.0)	36 (43.4)
Lipid formulation	5^e^ (8.5)	1 (20.0)	10^e^ (11.9)	1 (10.0)	15^e^ (10.5)	2 (13.3)
Ketoconazole only	1 (1.7)	1 (100.0)	0 (0)	NA	1 (0.7)	1 (100.0)
Posaconazole only	0 (0)	NA	1 (1.2)	0 (0)	1 (0.7)	0 (0)
Combination	3^f^ (5.1)	2 (66.7)	10^g^ (11.9)	1 (10.0)	13 (9.1)	3 (23.1)
Other	1^h^ (1.7)	0 (0)	0 (0)	NA	1 (0.7)	0 (0)

Abbreviation: NA, not applicable.

a Percentages are numbers of patients with or without treatment who died/total numbers with or without treatment.

b One patient with mucormycosis of the maxilla underwent surgery without antifungal treatment.

c Two patients with thigh and leg mucormycosis underwent surgery without antifungal treatment; both survived.

d Two patients were treated with deoxycholate amphotericin B sequentially followed by posaconazole.

e One patient each from Brazil and Argentina was treated with lipid formulations of amphotericin B sequentially followed by posaconazole.

f Includes lipid formulation of amphotericin B + voriconazole (2) and deoxycholate amphotericin B + posaconazole (1).

g Includes deoxycholate amphotericin B + posaconazole (2), deoxycholate amphotericin B + itraconazole (2), deoxycholate amphotericin. B + voriconazole (1), deoxycholate amphotericin B + ketoconazole (1), lipid formulation of amphotericin B + posaconazole (2) and lipid formulation of amphotericin B + caspofungin (2).

h Patient with right fourth finger mucormycosis caused by *Mucor hiemalis* was treated with potassium iodide.

Amphotericin B was the most widely used antifungal treatment in both Brazil (monotherapy: 44 patients, 74.6%; combination therapy: 3 patients, 5.1%) and the other countries (monotherapy: 54 patients, 64.3%; combination therapy: 10 patients, 11.9%; Table 4). Amphotericin B was mostly administered as the deoxycholate formulation. However, increased use of lipid formulations was noted over time, with only two patients receiving lipid formulations prior to 2010 (1 patient receiving combination therapy in French Guiana in 2008 and 1 patient receiving monotherapy in Brazil in 2009), compared with 19 cases receiving lipid formulations as monotherapy or combination therapy in 2010 or later. Substantial variation in mortality rates was observed between the different formulations of amphotericin B (deoxycholate formulation: 43.4%, lipid formulations: 13.3%; Table 4).

## 4 DISCUSSION

To our knowledge, this is the first review of cases of mucormycosis in South America, collating data from 143 patients in the published literature. Our results show a rise in the number of reported cases seen since 1970. Potential reasons for this increase include an improvement in disease awareness and diagnosis, a possible reduction in reluctance to report infections with a fatal outcome, and increasing ease of publication.

Patients identified in our literature searches were of comparable age (median 40 years) to those reported from a global review (median 39 years).^11^ However, our median patient age was younger than that observed in Europe‐wide and global studies (median 50 and 49 years, respectively),^4^, ^10^ or studies in specific countries such as France and Italy (median 58 and 60 years, respectively).^9^, ^19^ Most cases in South America occurred in the 18‐44 years and 45‐64 years age groups (74.8%). Additionally, most patients were males (61.5%), which is also consistent with findings (60%‐70% male) across studies from other regions.^4^, ^9^, ^10^, ^11^, ^14^, ^19^

Overall, our data on underlying conditions and mortality are broadly consistent with published studies.^4^, ^5^, ^7^, ^10^, ^11^ Diabetes mellitus was the most frequent underlying condition in South America, followed by penetrating trauma or burns, malignancy and SOT. This is largely consistent with previous reports from other regions.^3^, ^4^, ^6^, ^11^, ^15^ Despite an increase in the prevalence of diabetes in South America during the study period,^27^ the proportion of cases caused by diabetes in this study remained relatively constant over time. This may reflect improved overall care of diabetic patients in the region and consequently less patients presenting with diabetic ketoacidosis that predisposes to mucormycosis.

The large proportion of patients with penetrating trauma/burns aligns with the predominance of the 18‐44 years and 45‐64 years age groups in the patient population in South America. Workplace and traffic accidents may be more common in people of working age, and the association of accidents with penetrating trauma/burns is supported by a long‐term study in France.^28^ Many of the cases associated with penetrating trauma were associated with natural disasters such as volcanic eruptions, particularly in Colombia.^28^, ^29^ This link is supported by similar observations in other regions and suggests that prophylaxis against mucormycosis might be considered after natural disasters.

In patients identified in our literature searches, mortality was high (48.3%), but was within the range (40%‐70%) reported in studies from other regions.^4^, ^7^, ^10^, ^11^ Mortality among patients with malignancy was relatively high (11/19; 57.9%), and a mortality rate of 100% was observed in the three allogeneic HSCT recipients. Of note, while mortality was high with diabetes mellitus and penetrating trauma/burns (45.0% and 37.9%, respectively), it was even higher (58.5%) for more profoundly immunocompromised patients, that is patients receiving corticosteroids, patients with malignancy and transplant recipients. This confirms previous work^4^, ^11^ and also our personal experience in this area, which indicates that the primary determinant of outcome is the immune status of the host.

The most frequent sites of infection were, in order, rhino‐sino‐orbito‐cerebral, skin/soft tissues and pulmonary. Diabetes mellitus and malignancy were mostly associated with rhino‐sino‐orbito‐cerebral infections, while penetrating trauma or burns were mostly associated with skin and soft tissue infections. This is also mostly consistent with previous reports from other regions,^3^, ^4^, ^6^, ^15^ although malignancies have been associated with pulmonary infections in previous reports.^11^ Further analysis of the types of malignancies observed may clarify the cause of this discrepancy.

Pulmonary infections were more frequently observed in Brazil (23.7%) by a large margin compared to the other countries (3.6%). However, the underlying conditions were diverse, providing little basis for understanding the reason for this difference. Differences in infection site and mortality between Brazil and the other countries may be associated with factors that are beyond the scope of this review.

Across the entire period reviewed, approximately one in five patients in South America received no treatment. However, the proportion of patients receiving treatment increased over time, perhaps reflecting improvements in general medical care, including diagnostic resources and the availability of antifungal agents. Treatment typically involved the use of an antifungal agent, sometimes in combination with surgery. The most widely used antifungal agent was amphotericin B deoxycholate. While the addition of surgery was less frequent in Brazil, this may have been due to the lower incidence of skin and subcutaneous involvement there than in the other countries, resulting in decreased usefulness of debridement as a therapeutic option. The decision to perform surgery in mucormycosis cases varies with severity of illness and site of infection (eg sinuses and skin vs lungs) and depends on recognition by clinicians and surgeons of the impact of surgery on the outcome of mucormycosis. The nature of this manuscript (a review of published cases) does not allow us to elaborate on these differences.

Previous studies suggest that optimal outcomes in patients with mucormycosis may be achieved with a combination of lipid formulations of amphotericin B and surgery.^4^ In our review, mortality in patients receiving no treatment was >90%, but fell dramatically in patients treated with antifungals. As with previous studies, mortality rates were considerably lower with lipid formulations of amphotericin B compared with amphotericin B deoxycholate. More widespread use of effective antifungal therapy may improve mortality rates, but may currently be limited by the high cost of lipid formulations of amphotericin B.

Given the prominence of diabetes mellitus as an underlying cause of mucormycosis across South America, it is notable that despite improvements in treatment options for diabetes, the incidence of diabetes as a risk factor has not changed over time. Therefore, improved control of diabetes should result in a decrease in the overall incidence of mucormycosis in the region. More generally, our data on patterns of infection sites occurring in association with particular underlying conditions might offer targets for monitoring and prophylactic treatment in vulnerable individuals in South America, which may help to reduce infection rates. In addition, further confirmation in large data sets is required on the causes of differences between countries, such as the much higher association between penetrating trauma/burns and mucormycosis in South American countries other than Brazil, putatively related to a higher frequency of natural disasters. Such data will aid preventative efforts in the region.

*Rhizopus* and *Mucor* species were found to be the most frequently isolated organisms, but identification of the agent was not performed in half of the cases.

This study has various limitations, including a low number of cases overall in a region with extremely high environmental, socioeconomic and ethnic diversity, making it difficult to draw detailed conclusions. In addition, it is not possible to discern the causes for some of the patterns observed, such as the varying mortality rates with different sites of infection or with different underlying conditions. These issues may be addressed in future research as more cases are reported.

This case review identifies patterns of infection in South America that are of value to physicians in the region and may aid in formulating preventative efforts against mucormycosis. It also identifies gaps in current practice that, if addressed, could improve treatment across the region. Overall, increasing use of antifungals and surgery has much improved the prognosis of mucormycosis. However, mortality remains high, particularly in patients where surgery is not performed.^7^, ^12^, ^14^, ^30^ A combination of new diagnostic technologies, optimised use of available antifungal options, development of new antifungal agents and more aggressive public health policies may help to reduce mortality rates from mucormycosis in South America.

## CONFLICT OF INTEREST

ME and KH are employees of Basilea Pharmaceutica International Ltd. MN has received honoraria from AbbVie, Astellas, Gilead, Janssen, Merck, Pfizer, Teva and United Medical.

## AUTHOR CONTRIBUTIONS

K.H. and M.E. conceived the idea; K.H. collected and analysed the data; K.H. and M.N. led the writing.
