# Supplementary material for: Mucormycosis in South America: A review of 143 reported cases
Source: Mycoses. 2019 Jul 11;62(9):730–8. doi: 10.1111/myc.12958 (PMC6852100; doi:10.1111/myc.12958)
Supplement: Supplementary file 1 [file MYC-62-730-s001.docx]

# Appendices

## Supplementary Table 1. Use of antifungal treatment combined with surgery according to underlying condition: all South American countries combined

| Site of infection | Antifungal treatment plus surgery  n/N (%) |
| --- | --- |
| Rhino-sino-orbito-cerebral | 36/64 (56.3) |
| Skin and soft tissues | 22/34 (64.7) |
| Other | 6/8 (75.0) |
| Gastrointestinal or peritoneum | 3/5 (60.0) |
| Disseminated infection | 2/12 (16.7) |
| Liver or kidneys | 1/3 (33.3) |
| Pulmonary | 1/17 (5.9) |
| **Total** | **71/143 (49.7)** |

## Supplementary Table 2. Treatment and outcome among identified cases of mucormycosis in South America based on site of infection

| Site of infection | Brazil | | Other South American countries | | Total | |
| --- | --- | --- | --- | --- | --- | --- |
|  | Incidence n (%) | Mortality n (%)^†^ | Incidence n (%) | Mortality n (%)^†^ | Incidence n (%) | Mortality n (%)^†^ |
| Skin and soft tissues | N=6 | 0 (0) | N=28 | 11 (39.3) | N=34 | 11 (32.4) |
| Antifungal only | 4 (66.7) | 0 (0) | 2 (7.1) | 2 (100.0) | 6 (17.6) | 2 (33.3) |
| Antifungal and surgery | 2 (33.3) | 0 (0) | 20 (71.4) | 5 (25.0) | 22 (64.7) | 5 (22.7) |
| No antifungal treatment | 0 (0) | 0 (0) | 6 (21.4) | 4 (66.7) | 6 (17.6) | 4 (66.7) |
| Other sites of infection | N=53 | 31 (58.5) | N=56 | 27 (48.2) | N=109 | 58 (53.2) |
| Antifungal only | 24 (45.3) | 16 (66.7) | 13 (23.2) | 7 (53.8) | 37 (33.9) | 23 (62.2) |
| Antifungal and surgery | 19 (35.8) | 5 (26.3) | 30 (53.6) | 7 (23.3) | 49 (45.0) | 12 (24.5) |
| No antifungal treatment | 10 (18.9) | 10 (100.0) | 13 (23.2) | 13 (100.0) | 23 (21.1) | 23 (100.0) |

^†^Percentages are numbers of patients with or without treatment who died/total numbers with or without treatment.

## References for Brazil cases

1. Abboud CS, Bergamasco MD, Baia CE, et al. Case report of hepatic mucormycosis after liver transplantation: successful treatment with liposomal amphotericin B followed by posaconazole sequential therapy. *Transplant Proc.* 2012; 44(8): 2501-2502.

2. Abreu B, Duarte ML, Santos LRD, Sementilli A, Figueiras FN. A rare case of gastric mucormycosis in an immunocompetent patient. *Rev Soc Bras Med Trop.* 2018; 51(3): 401-402.

3. Azevedo V, Felippe L, Silva L, Parchen C, Queiros Telles F. Pulmonary fungal infection with hyalohyphomycosis associated with zygomycosis and Actinomyces spp. in a patient with ankylosing spondylitis. *Revista Brasileira de Reumatologia.* 2009; 49(5): 630-637.

4. Basilio FM, Hammerschmidt M, Mukai MM, Werner B, Pinheiro RL, Moritz S. Mucormycosis and chromoblastomycosis occurring in a patient with leprosy type 2 reaction under prolonged corticosteroid and thalidomide therapy*. An Bras Dermatol.* 2012; 87(5): 767-771.

5. Benites BM, Fonseca FP, Parahyba CJ, Arap SS, Novis YA, Fregnani ER. Extensive Oral Mucormycosis in a Transplanted Patient. *J Craniofac Surg.* 2017; 28(1): e4-e5.

6. Borges VN, Medeiros S, Ziomkowski S, Machado A. Successful Treatment of Mucormycosis and Aspergillus sp. Rhinosinusitis in an Immunocompromised Patient. *Braz J Infect Dis*. 1998; 2(4): 209-211.

7. Brandao A, Marroni CA, Cerski CT, Gleisner AL, Zanotelli ML, Cantisani G. [Zygomycosis following liver transplantation in adults: report of three cases]. *Rev Soc Bras Med Trop*. 2003; 36(6): 729-733.

8. Carvalhal GF, Machado MG, Pompeo A, Saldanha L, Sabbaga E, Arap S. Mucormycosis presenting as a renal mass in a patient with the human immunodeficiency virus. *J Urol*. 1997; 158(6): 2230-2231.

9. Cirino CG. Mucormicose. *Rev Bras Clin Terap.* 1987; 16(5): 137-138.

10. Costa AR, Porto E, Tayah M, et al. Subcutaneous mucormycosis caused by Mucor hiemalis Wehmer f. luteus (Linnemann) Schipper 1973. *Mycoses.* 1990; 33(5): 241-246.

11. Cunha MA, Nery AF, Lima FP, et al. Rhinocerebral zygomycosis in a diabetic patient. *Rev Soc Bras Med Trop.* 2011; 44(2): 257-259.

12. Damante JH, Fleury RN. Oral and rhinoorbital mucormycosis: case report. *J Oral Maxillofac Surg.* 1998; 56(2): 267-271.

13. de Almeida Junior JN, Ibrahim KY, Del Negro GM, et al. Rhizopus arrhizus and Fusarium solani Concomitant Infection in an Immunocompromised Host. *Mycopathologia.* 2016; 181(1-2): 125-129.

14. de Fátima Andrade da Costa M, Rodrigues Cisneiros RM, Moreira Batista E, de Carvalho e Meira PH, Amanda Gabriela Siqueira, Cunha Rodrigues RR. A276 Mucormycosis in diabetic patients: case report and literature review. *Diabetology & Metabolic Syndrome.* 2018; 10(Supp 1): 27.

15. de Franco MF, Iriya K. [Orbital-rhino-cerebral phycomycosis associated with diabetic ketoacidosis. Report of a case]. *Rev Inst Med Trop Sao Paulo.* 1970; 12(5): 354-363.

16. de Medeiros CR, Bleggi-Torres LF, Faoro LN, et al. Cavernous sinus thrombosis caused by zygomycosis after unrelated bone marrow transplantation. *Transpl Infect Dis.* 2001; 3(4): 231-234.

17. de Oliveira-Neto MP, Da Silva M, Fialho Monteiro PC, et al. Cutaneous mucormycosis in a young, immunocompetent girl. *Med Mycol.* 2006; 44(6): 567-570.

18. Deboni MC, Pozzani VR, Lisboa T, Hiraki K, Viplich R, Naclerio-Homem MG. Mucormycosis in an immunocompetent patient: follow-up of 1 year after treatment. *Acta Otolaryngol.* 2006; 126(9): 993-996.

19. Ferreira FC, Ishii CK, Kusabara AA, Godinho JVV, Hida RY. Fungal endophthalmitis caused by Zygomycetes after phacoemulsification. *JCRS Online Case Reports.* 2018; 6(3): 43-46.

20. Grellet M, Colatemina JF, Figueiredo Dias F, Figueiredo DJ, Garcia Jaeger WL. [Mucormycosis of the paranasal sinus]. *BJORL.* 1977; 43(1): 18-25.

21. Guerreiro CA, Nobrega JP, Carvalho MP. [Orbito-rhino-cerebral phycomycosis (mucormycosis): report of a case]. *Arq Neuropsiquiatr.* 1980; 38(1): 99-105.

22. Haron ES, Leirner AA, Carneiro PC, Pereira VG. [Gastric mucormycosis associated with antibiotic and corticosteroid therapy]. *Rev Hosp Clin Fac Med Sao Paulo.* 1981; 36(2): 89-90.

23. Lauand F, Lia RC, Marcantonio E, Gullo N, Paino MA. [Mucormycosis of the maxilla: case report]. *Ars Curandi Odontol.* 1977; 4(1): 22-27.

24. Lopes JO, de Mello ES, Klock C. Mixed intranasal infection caused by Fusarium solani and a zygomycete in a leukaemic patient. *Mycoses.* 1995; 38(7-8): 281-284.

25. Lopes JO, Pereira DV, Streher LA, Fenalte AA, Alves SH, Benevenga JP. Cutaneous zygomycosis caused by Absidia corymbifera in a leukemic patient. *Mycopathologia.* 1995; 130(2): 89-92.

26. Mayayo E, Klock C, Goldani LZ, Monteiro AC, Capilla J. [Mixed fungal infection in a diabetic patient]. *Rev Iberoam Micol.* 2010; 27(3): 140-143.

27. Moreira J, Ridolfi F, Almeida-Paes R, Varon A, Lamas CC. Cutaneous mucormycosis in advanced HIV disease. *Braz J Infect Dis.* 2016; 20(6): 637-640.

28. Neto FM, Camargo PC, Costa AN, et al. Fungal infection by Mucorales order in lung transplantation: 4 case reports. *Transplant Proc.* 2014; 46(6): 1849-1851.

29. Oliveira FR, Couto NG, Bastos JO, Colleti JJ, Carvalho WB. Abdominal mucormycosis in a child: a case report. *Rev Soc Bras Med Trop.* 2016; 49(6): 796-198.

30. Oliveira JS, Kerbauy FR, Colombo AL, et al. Fungal infections in marrow transplant recipients under antifungal prophylaxis with fluconazole. *Braz J Med Biol Res.* 2002; 35(7): 789-798.

31. Passos XS, Sales WS, Maciel PJ, Costa CR, Ferreira DM, do Silva MR. Nosocomial invasive infection caused by Cunninghamella bertholletiae: case report. *Mycopathologia.* 2006; 161(1): 33-35.

32. Paulo De Oliveira JE, Milech A. A fatal case of gastric mucormycosis and diabetic ketoacidosis. *Endocr Pract.* 2002; 8(1): 44-46.

33. Queiroz-Telles F, Coelho A, Porto E, et al. Subcutaneous mucormycosis caused by Rhizopus oryzae: probable nosocomial acquired infection. *Revista do Instituto de Medicina Tropical de São Paulo.* 1985; 27(4): 201-206.

34. Raymundo IT, Araujo BG, Costa Cde C, Tavares JP, Lima CG, Nascimento LA. Rhino-orbito-cerebral mucormycosis. *Braz J Otorhinolaryngol.* 2009; 75(4): 619.

35. Ribeiro EF, dos Santos VM, Paixao GT, Cruz LR, Danilow MZ, Campos VF. Mucormycosis in a patient with acute myeloid leukemia successfully treated with liposomal amphotericin B associated with deferasirox and hyperbaric oxygen. *Mycopathologia.* 2013; 175(3-4): 295-300.

36. Ribeiro LC, Wanke B, da Silva M, et al. Mucormycosis in Mato Grosso, Brazil: a case reports, caused by Rhizopus microsporus var. oligosporus and Rhizopus microsporus var. rhizopodiformis. *Mycopathologia.* 2012; 173(2-3): 187-192.

37. Santana NOR, Pinheiro GB, Kehrle HM, Faria IPd, Estrella CN. [Mucormycosis of the paranasal sinuses and orbit in a immunocompetent patient: a case report and review of the literature]. *Rev Bras Otorrinolaringol.* 2001; 67(5): 727-730.

38. Severo C, de Mattos Oliveira F, Dreher R, Teixeira Z, da Silva Porto N, Thomaz Londero A. Zygomycosis: A report of eleven cases and a review of the Brazilian. *Rev Iberoam Micol.* 2002; 19: 52-56.

39. Severo LC, Job F, Mattos TC. Systemic zygomycosis: nosocomial infection by Rhizomucor pusillus. *Mycopathologia.* 1991; 113(2): 79-80.

40. Timoteo CA, Correa AP, Zorzi Colete J, Marcondes Aranega A, Junior IR. Survival Without Neurological Impairment of a Patient With Rhino-Orbito-Cerebral Zygomycosis. *J Craniofac Surg.* 2016; 27(4): e376-378.

41. Tuder RM. Myocardial infarct in disseminated mucormycosis: case report with special emphasis on the pathogenic mechanisms. *Mycopathologia.* 1985; 89(2): 81-88.

42. Wittig EO, Cat I, Abdala H, Kasting G. [Meningoencephalitis and mucormycosis]. *Arq Neuropsiquiatr.* 1973; 31(2): 151-155.

43. Xavier S, Polacow Korn G, Granato L. Rhinocerebral mucormycose: Case presentation and literature review. *Rev Bras Otorrinolaringol.* 2004; 70(5): 710-714.

## References for cases from the other South American countries

1. Astegiano M-G, Alexenicer O, Elias GA, Fonseca S, Teglia O. Rhinocerebral mucormycosis: Importance of an early diagnosis. *Prensa Medica Argentina*. 1992; 79(7): 432-435.

2. Berthier M, Palmieri O, Lylyk P, Leiguarda R. Rhino-orbital phycomycosis complicated by cerebral abscess. *Neuroradiology*. 1982; 22(4): 221-224.

3. Blanchet D, Dannaoui E, Fior A, et al. Saksenaea vasiformis infection, French Guiana. *Emerg Infect Dis*. 2008; 14(2): 342-344.

4. Borel C. Mucormicosis. *Rev Otorrinolaringol Cir Cabeza y Cuello*. 1987; 47(1): 29-35.

5. Bourke P, Castro P, Rabagliati R, et al. Zygomycosis over-infection during voriconazole therapy for aspergillosis in a heart transplant patient, successfully treated with liposomal amphotericin and posaconazole. *Transpl Infect Dis*. 2012; 14(5): E56-59.

6. Bravo JH, Agudelo AM, Cortes A, Matta L. [Rhino-orbito-cerebral mucormycosis from dental origin: Case report]. *Biomedica*. 2018; 38(1): 27-31.

7. Bravo M, Ferrer S, Etchard M, Trujillo S. [Rhinocerebral mucormycosis. Report of four cases]. *Rev Med Chil*. 1999; 127(6): 712-718.

8. Caceres AM, Sardinas C, Marcano C, et al. Apophysomyces elegans limb infection with a favorable outcome: case report and review. *Clin Infect Dis*. 1997; 25(2): 331-332.

9. Cofre F, Villarroel M, Castellon L, Santolaya ME. [Successful treatment of a persistent rhino-cerebral mucormycosis in a pediatric patient with a debut of acute lymphoblastic leukemia]. *Rev Chilena Infectol*. 2015; 32(4): 458-463.

10. Cox J, Corona S, Donoso S. [Orbital and cerebral mucormycosis in 2 diabetic patients]. *Rev Med Chil*. 1985; 113(4): 341-344.

11. Davel G, Featherston P, Fernandez A, et al. Maxillary sinusitis caused by Actinomucor elegans*. J Clin Microbiol*. 2001; 39(2): 740-742.

12. Delfor Podesta L, Golfera H, Rivero C. [Phycomycosis and leukemia]. *Prensa Med Argent*. 1971; 58(1): 57-60.

13. Duque Fisher CS, Gonzalez Norena HD, Lopera Calderon FJ. [Otorhinolaryngological manifestations of mucormycosis. Study of 6 cases]. *Acta Otorrinolaringol Esp*. 1996; 47(4): 291-294.

14. Featherston PL, Fernandez G, Munguia H, Marin ME. [Mucormycosis: Study of five cases found in San Juan de Dios hospital and Sor Maria Ludovica hospital in La Plata, Argentina]. *Rev Argent Microbiol*. 1998; 30(4): 176-179.

15. Frey Gutierrez ME, Martinez GW, Alvarez Milan L, Acuna Vassallo JP, Trinajstic EM. [Mucormycosis cutanea- clinical case presentation souvenir etiopatogenia diagnosis and treatment]. *Rev Fac Cien Med Univ Nac Cordoba*. 2016; 73(4): 302-305.

16. Gamarra S, Chaves MS, Cabeza MS, et al. Mucormycosis outbreak due to Rhizopus microsporus after arthroscopic anterior cruciate ligament reconstruction surgery evaluated by RAPD and MALDI-TOF Mass spectrometry. *J Mycol Med*. 2018; 28(4): 617-622.

17. Gonzalez Cueto D, Guman N. [Disseminated mucormycosis. Presentation of two cases]. *Medicina (B Aires)*. 1968; 28(5): 286-289.

18. Hurtado R, Spinner R, Pozo S, Veit O. [Cerebral mucormycosis]. *Rev Med Chil*. 1965; 93(4): 179-182.

19. Luna JD, Ponssa XS, Rodriguez SD, Luna NC, Juarez CP. Intraconal amphotericin B for the treatment of rhino-orbital mucormycosis. *Ophthalmic Surg Lasers*. 1996; 27(8): 706-708.

20. Marco del Pont J, De Cicco L, Gallo G, Llera J, De Santibanez E, D'Agostino D. Hepatic arterial thrombosis due to Mucor species in a child following orthotopic liver transplantation. *Transpl Infect Dis*. 2000; 2(1): 33-35.

21. Mata-Essayag S, Magaldi S, de Capriles CH, Henao L, Garrido L, Pacillo V. Mucor indicus necrotizing fasciitis. *Int J Dermatol*. 2001; 40(6): 406-408.

22. Patino JF, Castro D, Valencia A, Morales P. Necrotizing soft tissue lesions after a volcanic cataclysm. *World J Surg*. 1991; 15(2): 240-247.

23. Patino JF, Mora R, Guzman MA, Rodriguez-Franco E. Mucormycosis: a fatal case by Saksenaea vasiformis. *World J Surg*. 1984; 8(3): 419-422.

24. Pena CE, Dorado JA. [Mucormycosis (phycomycosis) in Colombia. Presentation of 2 cases]. *Rev Fac Med Univ Nac Colomb*. 1965; 33(4): 205-211.

25. Pinto ME, Manrique HA, Guevara X, Acosta M, Villena JE, Solis J. Hyperglycemic hyperosmolar state and rhino-orbital mucormycosis. *Diabetes Res Clin Pract*. 2011; 91(2): e37-39.

26. Pisarevsky AA, Plumet Garrido J, Mariash NC, Cordini G, Tiraboschi N, Petrucci EA. [Rhinosinusal mucormycosis]. *Medicina (B Aires)*. 2011; 71(4): 377.

27. Poch GF, Frugoni R, Ara C, Breglia M, Pascucelli H. [Thrombophlebitis of the cavernous sinus caused by mucormycosis. Report of a case]. *Prensa Med Argent*. 1969; 56(26): 1304-1305.

28. Relloso S, Romano V, Landaburu MF, et al. Saksenaea erythrospora infection following a serious sailing accident. *J Med Microbiol*. 2014; 63(Pt 2): 317-321.

29. Riera F, Marangoni LD, Allende BL, et al. [Mucormycosis. Clinical cases and update]. *Rev Fac Cien Med Univ Nac Cordoba*. 2014; 71(4): 192-198.

30. Rodriguez JY, Morales-Lopez SE, Rodriguez GJ, et al. Necrotizing fasciitis caused by Apophysomyces variabilis in an immunocompetent patient. *Med Mycol Case Rep*. 2018; 20: 4-6.

31. Rodriguez JY, Rodriguez GJ, Morales-Lopez SE, Cantillo CE, Le Pape P, Alvarez-Moreno CA. Saksenaea erythrospora infection after medical tourism for esthetic breast augmentation surgery. *Int J Infect Dis*. 2016; 49: 107-110.

32. Ruiz CE, Arango M, Correa AL, Lopez LS, Restrepo A. [Necrotizing fasciitis in an immunocompetent patient caused by Apophysomyces elegans]. *Biomedica*. 2004; 24(3): 239-251.

33. Strazza LR, Guzman J, Ghelli RE, et al. [Pulmonary mucormycosis in a patient with diabetes mellitus: favorable evolution under medical treatment]. *Rev Fac Cien Med Univ Nac Cordoba*. 2007; 64(3): 93-98.

34. Tager FM, Zaror CL, Martinez DP. [Cutaneous mucormycosis in an immunocompromised patient]. *Rev Chilena Infectol*. 2012; 29(1): 101-107.

35. Tapia EO, Chahin AC, Concha FC. [Primary cutaneous mucormycosis: two case reports and review of the literature]. *Rev Chilena Infectol*. 2011; 28(3): 269-273.

36. Tiraboschi I, Bravo M, Fernandez N, Stecher D, Melero M, Lasala M. [Mucormycosis. An emergent mycosis]. *Medicina (B Aires)*. 2012; 72(1): 23-27.

37. Tobon AM, Arango M, Fernandez D, Restrepo A. Mucormycosis (zygomycosis) in a heart-kidney transplant recipient: recovery after posaconazole therapy. *Clin Infect Dis*. 2003; 36(11): 1488-1491.

38. Torres-Damas W, Yumpo-Cardenas D, Mota-Anaya E. [Coinfection of rhinocerebral mucormycosis and sinus aspergillosis]. *Rev Peru Med Exp Salud Publica*. 2015; 32(4): 813-817.

39. Tristano A, Chollet ME, Willson M, Troccoli M. [Mucormycosis. Report of 3 cases]. *Invest Clin*. 2002; 43(3): 183-190.

40. Vainrub B, Macareno A, Mandel S, Musher DM. Wound zygomycosis (mucormycosis) in otherwise healthy adults. *Am J Med*. 1988; 84(3 Pt 1): 546-548.

41. Vega W, Orellana M, Zaror L, Gene J, Guarro J. Saksenaea vasiformis infections: case report and literature review. *Mycopathologia*. 2006; 162(4): 289-294.
